# The changing landscape of bluetongue in northern Europe

**DOI:** 10.1093/jme/tjag071

**Published:** 2026-05-25

**Authors:** Marion England, Thomas Balenghien, Carrie Batten, Emmanuel Bréard, Ilse De Leeuw, Nick De Regge, Maxime Duhayon, Claire Garros, Pachka Hammami, Melle Holwerda, Archie Murchie, Christopher Sanders, Mathilde Uiterwijk, René van den Brom, Eva Veronesi, Simon Gubbins

**Affiliations:** The Pirbright Institute, Woking, UK; CIRAD, UMR ASTRE, Montpellier, France; ASTRE, Univ Montpellier, CIRAD, INRAE, Montpellier, France; The Pirbright Institute, Woking, UK; UMR1161 Virologie, ANSES, INRAE, Ecole Nationale Vétérinaire d’Alfort, Université Paris-Est, Maisons-Alfort, France; National Reference Laboratory for Bluetongue, Service of Exotic and Vector-Borne Diseases, Sciensano, Brussels, Belgium; National Reference Laboratory for Bluetongue, Service of Exotic and Vector-Borne Diseases, Sciensano, Brussels, Belgium; CIRAD, UMR ASTRE, Montpellier, France; ASTRE, Univ Montpellier, CIRAD, INRAE, Montpellier, France; CIRAD, UMR ASTRE, Montpellier, France; ASTRE, Univ Montpellier, CIRAD, INRAE, Montpellier, France; CIRAD, UMR ASTRE, Montpellier, France; ASTRE, Univ Montpellier, CIRAD, INRAE, Montpellier, France; Wageningen Bioveterinary Research (WBVR), Department of Virology, Lelystad, The Netherlands; Sustainable Agri-Food Sciences Division, Agri-Food and Biosciences Institute, Belfast, UK; The Pirbright Institute, Woking, UK; Centre for Monitoring of Vectors (CMV), Netherlands Institute for Vectors, Invasive Plants and Plant Health, Netherlands Food and Consumer Product Safety Authority (NVWA), Wageningen, The Netherlands; Royal GD, Deventer, The Netherlands; Institute Microbiology, Department for Environment Constructions and Design, University of Applied Sciences and Arts of Southern Switzerland (SUPSI), Mendrisio, Switzerland; The Pirbright Institute, Woking, UK

**Keywords:** Bluetongue, *Culicoides*, Europe, climate change, globalization

## Abstract

Bluetongue was historically a disease of the tropics and subtropics, but over the past 20 yr has emerged in temperate regions such as northern Europe. Multiple serotypes of bluetongue virus (BTV) have been introduced into northern Europe, with some becoming enzootic, most notably 3, 4, and 8 in France. This review describes the step-change in the occurrence of bluetongue across northern Europe with a focus on changes since the re-emergence of BTV-8 in France in 2015. Five countries in northern Europe are specifically discussed—Belgium, France, the Netherlands, the United Kingdom, and Switzerland. These countries have all been significantly affected by the widespread outbreak of BTV-3, which emerged in 2023. Here, the latest data on case numbers, disease and *Culicoides* surveillance, and the current state of play for each country are presented. The latest research into vector competence, transmission studies, and advances in vaccination and vector control, relevant to northern Europe, are summarized. Finally, climate change and globalization are critically examined as reasons for the epidemiological changes in BTV occurrence across Europe.

## Introduction

Bluetongue virus (BTV) is an *Orbivirus* (family Sedoreoviridae) that is primarily transmitted by *Culicoides* biting midges and causes bluetongue disease in wild and domestic ruminants (Mellor *et al.*, 2000). Historically a disease of the tropics and subtopics, bluetongue has more recently impacted temperate regions, including northern Europe. Although multiple BTV serotypes (BTV-1, BTV-2, BTV-4, BTV-9, and BTV-16) were circulating in southern Europe from the late 1990s onward, north-western Europe remained free of BTV until the 2000s (Saegerman et al. 2008). Between 1998 and 2006, Mediterranean Europe experienced 5 different BTV serotypes, and taken together, this period is reflective of a step-change in the occurrence of bluetongue disease across Europe. Emergence and spread of BTV in southern and eastern Europe, both before and after 1998, have been previously discussed, along with the unprecedented BTV serotype 8 (BTV-8) emergence in northern Europe in 2006 (Purse et al. 2005, Mellor et al. 2008, Saegerman et al. 2008, Wilson and Mellor 2009).

The origin and route of incursion of BTV-8 into northern Europe remains unknown, but this sustained outbreak demonstrated the ability of BTV to be transmitted by Palearctic species of *Culicoides*, in the absence of the traditional Afro-Asian vector species, *Culicoides imicola* Kieffer (Carpenter et al. 2009, Vanbinst et al. 2009), and that the virus was able to overwinter in temperate regions. At the time of the BTV-8 outbreak in 2007, BTV-1 was identified in southern Europe, spreading up through France during 2008. Between 1998 and 2019, 9 different BTV serotypes were detected in mainland Europe (data retrieved from World Organisation for Animal Health, WOAH), with several strains detected for some serotypes, alongside the novel orthobunyavirus, Schmallenberg virus (SBV), which emerged in 2011, and is also spread by *Culicoides* (Beer et al. 2013, Barber et al. 2018). Epizootic haemorrhagic disease virus (EHDV, serotype 8), an *Orbivirus* also transmitted by *Culicoides*, was reported in mainland Europe (Spain) for the first time in 2022 (Jiménez-Cabello et al. 2023). It spread rapidly north- and west-wards to France and Portugal throughout 2023 and 2024 (Gondard et al. 2024), followed by markedly fewer detections in 2025.

**Fig. 1.**
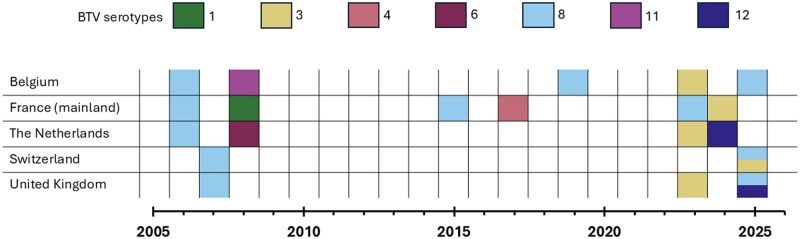
Historical timeline of bluetongue virus serotypes in Belgium, mainland France, the Netherlands, United Kingdom, and Switzerland from 2005 to present. Serotypes are represented in green (BTV-1), yellow (BTV-3), red (BTV-4), burgundy (BTV-6), blue (BTV-8), dark pink (BTV-11), and dark blue (BTV-12). Dates represent when serotypes first emerged or re-emerged within a country. Atypical serotypes and isolated imports with no evidence of onward transmission are excluded. Disease reports retrieved from WOAH.

**Fig. 2.**
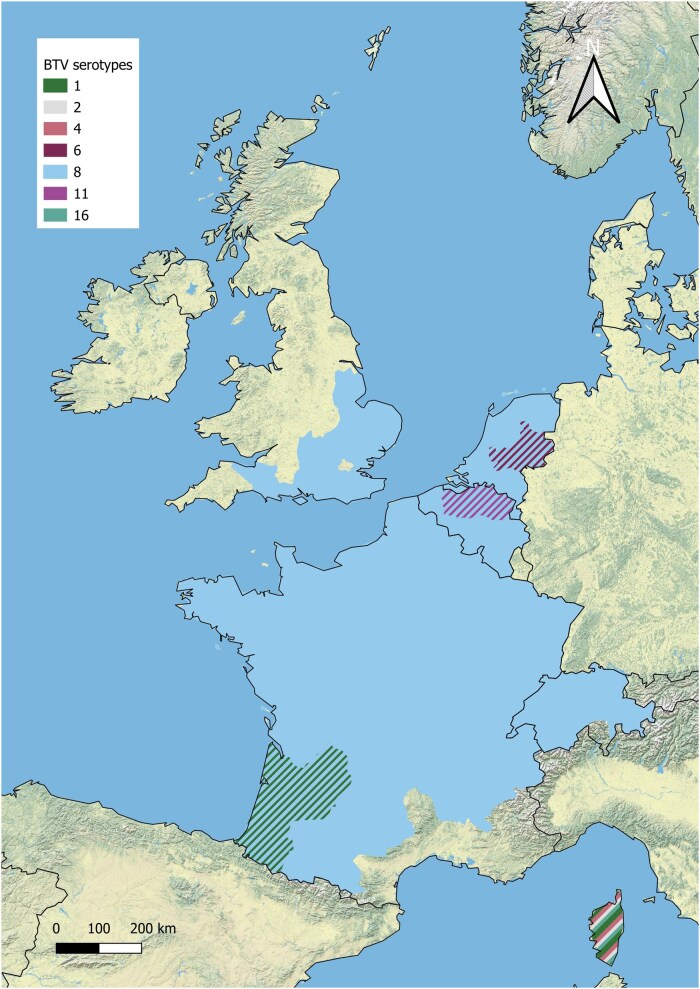
Map of northern Europe showing BTV serotypes that caused disease outbreaks in Belgium, France, the Netherlands, Switzerland and the United Kingdom between 2000 and 2014. Disease reports retrieved from WOAH, serotypes declared as enzootic may extend further than shown.

**Fig. 3.**
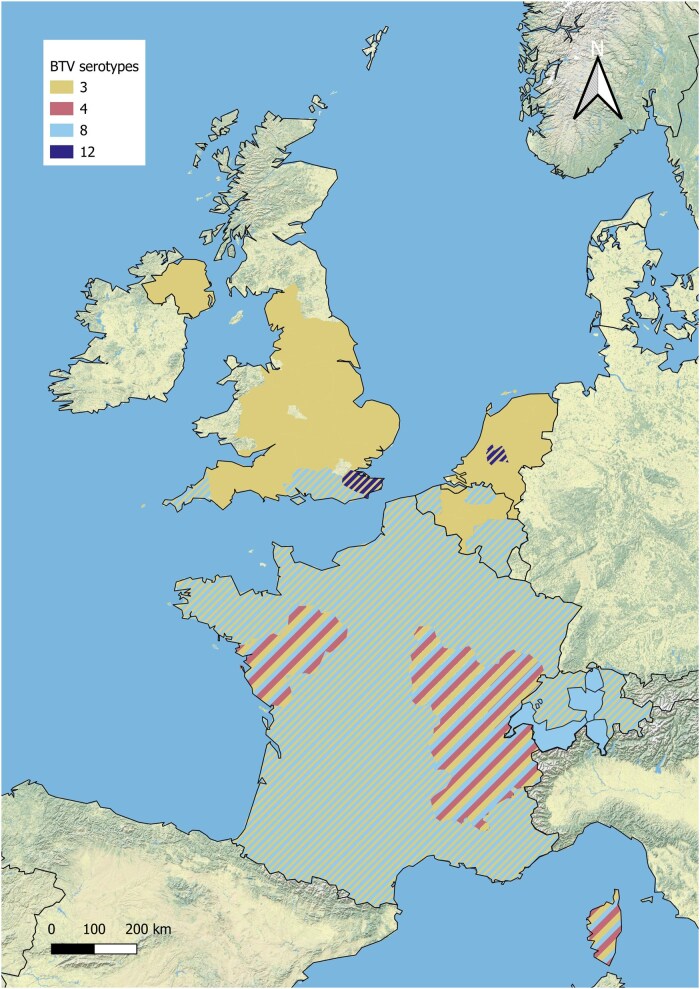
Map of northern Europe showing BTV serotypes that caused disease outbreaks in Belgium, France, the Netherlands, Switzerland and the United Kingdom between 2015 and 2026. Disease reports retrieved from WOAH, serotypes declared as enzootic may extend further than shown.

The purpose of this review is to provide a situational update on BTV epidemiology across northern Europe, with a focus on Belgium, France, the Netherlands, Switzerland and the United Kingdom. These 5 countries have experienced multiple incursions of BTV serotypes over the last 10 (Figs 1 and 2, data retrieved from WOAH) to 20 yr (Figs 1 and 3, data retrieved from WOAH). Here, we present a brief history of bluetongue in 5 countries of northern Europe, specifically focussing on the challenges faced since the re-emergence of BTV in northern Europe in 2015. We provide an overview of surveillance and monitoring within our countries of focus and highlight key advancements in BTV and *Culicoides* research. We provide insight into the increasing burden of BTV in northern Europe in the context of our changing climate and identify research gaps and future directions to improve our preparedness and response to bluetongue outbreaks and related *Culicoides-*borne arboviruses.

## BTV in Europe Pre-2015

### The Netherlands

In August 2006, the first outbreak of bluetongue in northern Europe occurred in the south-eastern part of the Netherlands. The virus responsible was identified as BTV-8 (Maan et al. 2008), and by the end of the first year of the outbreak, 185 holdings were affected in the Netherlands. BTV-8 overwintered and caused morbidity and mortality in 2007, mainly in sheep but also in cattle, and by the end of 2007, BTV-8 was notified on 6,442 holdings. In 2008, a large voluntary vaccination campaign with inactivated BTV-8 vaccines was initiated. The campaign resulted in a limited number of BTV-8 cases in 2008 (60 holdings) in the northern part of the Netherlands, and the last clinical cases in unvaccinated ruminants of the 2006 to 2008 BTV-8 outbreak. Also in 2008, 2 other BTV serotypes were detected in the Netherlands: BTV-6, genotyped as a live-attenuated vaccine strain, and 1 case of BTV-1, which was the result of cattle imported from Spain, where that specific serotype was circulating (van Rijn et al. 2012). In 2012, the Netherlands became officially free of bluetongue.

### France

The history of bluetongue in France, from 2000 to 2018, has been previously reviewed by Kundlacz et al. (2019). Bluetongue serotype 2 (BTV-2) was the first serotype to emerge in France (Corsica) in October 2000, just after the first detection of *C*. *imicola* in September 2000 in Corsica (Delécolle and de La Rocque 2002). Subsequently, both BTV-4 and BTV-16 were detected in Corsica in 2003 and 2004, respectively. In 2006, when BTV-8 emerged in northern Europe, France (mainland) was initially only marginally affected, with just 7 affected holdings along the border with Belgium. However, throughout 2007 and 2008, there was significant transmission by autochthonous *Culicoides* species across the whole French territory, resulting in 15,253 affected holdings in 2007 and 23,959 affected holdings in 2008. Simultaneously, BTV-1 was introduced in the Basque Country from southern Spain, with significant circulation (4,339 affected holdings in 2008), mainly in south-western France. Following vaccination campaigns, France regained BTV-free status in 2012. In 2013, BTV-1 was detected in Corsica, and in 2014, a novel atypical BTV serotype, BTV-27, transmitted directly between goats, was found in Corsica.

### UK

The first case of BTV-8 was detected in the United Kingdom in September 2007, and after a voluntary vaccination programme in the spring of 2008, no new cases were detected during the 2008 transmission season. Some cases were detected in early 2008 through pre-movement testing, but these were considered to be infections from the previous 2007 transmission season, resulting in a total of 125 BTV-8-affected holdings for the United Kingdom during this outbreak. Pregnant heifers, which were RT-PCR-negative, were imported into Northern Ireland from the Netherlands in January 2008 and gave birth to RT-PCR-positive calves, providing evidence of transplacental transmission. Two further cows from the same herd were considered to have been infected by direct contact with BTV-8-infected placentas (Menzies et al. 2008).

### Belgium

Bluetongue was first identified in Belgium in 2006, 1 d after the confirmation of disease in the Netherlands (Toussaint et al. 2007). By the end of the 2006 transmission season, Belgium had reported 695 affected holdings, confirming that BTV-8 was able to spread efficiently after its introduction. The detection of new cases at the start of the 2007 vector season further indicated that the virus had successfully overwintered in the region, leading to a much larger epidemic that year, with 6,870 affected holdings reported nationwide (Méroc et al. 2009). When inactivated BTV-8 vaccines became available in 2008, Belgium implemented a nationwide vaccination campaign, which rapidly reduced virus circulation. Subsequent sentinel RT-PCR surveillance provided no evidence of BTV-8 circulation in 2010, consistent with the absence of reported clinical cases and RT-PCR analyses indicating that spleen samples from aborted foetuses were negative after mid-April 2010 (Garigliany et al. 2011, Vangeel et al. 2012).

Additional serotypes were detected only sporadically. BTV-1 and BTV-6 were found exclusively in imported animals, with no evidence of establishment or onward transmission in Belgium (De Clercq et al. 2009). Limited circulation of BTV-11 occurred in Belgium in November 2008. Molecular analyses showed that genome segment 2 of the virus was identical to a South African live attenuated vaccine strain. Follow-up surveillance identified a small number of additional positives, corresponding to an estimated prevalence of 0.2% at animal level and approximately 3% to 4% at herd level. These findings indicate limited local transmission without evidence of sustained circulation or wider spatial spread, consistent with a transient, vaccine-derived introduction (De Clercq et al. 2009, Vandenbussche et al. 2015).

### Switzerland

The first confirmed detection of BTV-8 in livestock animals in Switzerland was reported on 27 October 2007 in Bettingen, located in the canton of Basel-Stadt, close to the French border (Hofmann et al. 2008a, Schwermer et al. 2008). In accordance with control measures implemented in other European countries affected by the epidemic, Switzerland introduced a nationwide compulsory vaccination program in 2008 aimed at reducing virus circulation (Schwermer et al. 2008, Bruckner et al. 2009). The campaign initially targeted cattle, sheep, and goats for a period exceeding three months. From 2009 onwards, mandatory vaccination was limited to cattle and sheep, while vaccination of goats became voluntary. Vaccination coverage reached 76.8% in 2008, increased to 88.7% in 2009, and subsequently declined to 70.8% in 2010. From 2011 onwards, vaccination against BTV-8 was no longer mandatory and remained voluntary for all livestock species. Between 2007 and 2011, a cumulative total of 76 affected holdings had been reported in Switzerland (2007: 5, 2008: 35, 2009: 35, 2010: 1) with a total of 160 infected animals (Schorer and Schwermer 2012).

During BTV surveillance, a new *Orbivirus*, closely related to BTV but clearly different from known serotypes, was discovered in 2 goat herds from the Toggenburg region (Chaignat et al. 2009). Field investigations indicated widespread circulation in small ruminants with minimal or no apparent clinical signs. The genome sequence of this virus, named Toggenburg virus (TOV), revealed that it represented a new BTV serotype (BTV-25) (Hofmann et al. 2008b). Its detection revealed gaps in existing diagnostic and surveillance systems and raised unresolved questions regarding epidemiology, vector competence, and pathogenicity. Since its discovery, other atypical BTV serotypes have been identified in Europe, including from Sardinia (Italy) (Savini et al. 2017), Switzerland (Ries et al. 2021), Germany (Ries et al. 2020), and Corsica, as previously mentioned.

## BTV in Europe Post-2015

### The Netherlands

After 12 yr of absence, at the beginning of September 2023, a major BTV outbreak occurred again in the Netherlands. Sheep and cattle in the center of the country were the first affected and whole genome sequencing showed that the virus isolate belonged to BTV serotype 3 (Holwerda et al. 2024). In the 2023 season, approximately 6,000 holdings with clinical- and/or RT-PCR-positive ruminants were reported, mainly in the center and north of the Netherlands. In the 2024 season, despite widespread vaccination use, over 8,000 affected holdings with at least one BTV-positive animal were reported. The BTV-3 outbreak had a significant impact on the sheep population of the Netherlands, with high mortality and >50% case fatality rate in 2023 (Santman-Berends et al. 2024). Severe clinical signs were also observed in cattle, with significantly increased mortality, abortions and premature births (van den Brink et al. 2025). Infection with BTV-3 was also detected in a pregnant farm dog, suspected to have consumed material from a sheep that died from BTV (van den Brink et al. 2024). Similarly, clinical BTV-3 infection was also observed in four farm-associated pregnant dogs in Portugal in 2024, resulting in abortions and death (Barros et al. 2025). In 2025, bluetongue was not detected in the Netherlands, likely as a consequence of high immunity in the susceptible population after a large number of natural infections in 2023 and 2024, and widespread vaccination in 2024 and 2025. The origin and route of incursion of BTV-3 remain unknown.

Another BTV serotype was detected in the Netherlands in 2024: BTV-12 (van den Brom et al. 2025). This serotype had never been observed before in Europe and sequencing showed a potential common ancestor with a virus isolate that circulated in 2020 in Israel (Van den Brom et al. 2025). BTV-12 was only detected on 12 farms, and the within-farm prevalence on these farms was between 0.3% and 9.3%. BTV-12 did not appear to have overwintered, since no cases of BTV-12 were found in 2025.

### France

In 2015, BTV-8 re-emerged in central France, suggested to be the result of use of infectious frozen bull semen from the previous BTV-8 outbreak (Pascall et al. 2020). The clinical impact of this BTV-8 strain has been low. In 2017, BTV-4 was introduced in the east of France from Corsica through animal movements, although there has not been a significant onward spread. Since 2020, both BTV-8 and BTV-4 have been considered enzootic serotypes within France. However, a new strain of BTV-8 causing clinical disease was detected in 2023 and continues to be responsible for outbreaks in both mainland France and Corsica. The BTV-4 strain causing outbreaks in Corsica in 2023 was the same as that responsible for cases in 2016, 2017, and 2020 on the island, and is either the result of reintroduction from the continent or continuous silent circulation (Gondard et al. 2024).

In 2024, BTV-3 spread to northern France (Nord department that borders Belgium) with disease being confirmed on 5 August. In the first season of transmission (5 August 2024 to 1 June 2025), 10,813 BTV-3-affected holdings were recorded across France, and 17,038 holdings had animals infected (with clinical signs) with the new strain of BTV-8. Between June 2025 and January 2026, an additional 6,709 holdings reported BTV-3 and 3,338 holdings reported BTV-8. BTV-3 has now spread to most areas of mainland France. In Corsica, another strain of BTV-3 circulated in 2024, causing severe clinical disease in sheep. This BTV-3 strain is different from the Dutch strain; it is the product of a reassortment that took place in Sardinia, between an avirulent BTV-3 strain (whose origin is North Africa) and the BTV-4 strain that has been present in Sardinia for many years (Plebani et al. 2025).

### UK

Since the BTV-8 outbreak in 2007, there have been two occurrences of imports of BTV-infected animals (RT-PCR positive) into Great Britain (both infected with BTV-8) picked up through post-import testing. These did not result in onward transmission due to the time of year of import and swift measures taken at the affected premises, namely, culling of infected animals. In November 2018, a single heifer in a consignment of nine imported into Northern Ireland from France tested RT-PCR positive for BTV-8. The infected animal was culled, others in the shipment were isolated, and *Culicoides* activity was monitored on site. There was no subsequent onward transmission (Georgaki et al. 2019).

BTV-3 was detected for the first time in the United Kingdom in November 2023 at a beef farm in Kent (APHA 2023). A restricted zone was set up around the infected premises (IP), and all livestock ruminants within this zone were tested for BTV by RT-PCR. Following active surveillance, a total of 126 positive animals (119 cattle and seven sheep) at 73 holdings were detected across Kent, Norfolk, Suffolk, and Surrey (APHA 2024c). These resulted from 2 separate incursion events, with wind-borne transport of infected *Culicoides* from continental Europe highly likely to be the source of both incursion events.

Significantly, clinical signs associated with BTV infection were not observed in any of the 126 positive animals detected during the 2023/24 season. This is in stark contrast to the significant clinical impact experienced by the Netherlands in sheep as well as in cattle (Santman-Berends et al. 2024, van den Brink et al. 2025), and despite an identical virus strain that has since been experimentally shown to cause clinical disease in UK sheep (Newbrook et al. 2025). Several explanations have been suggested for this. One suggestion is that the majority of cases were in cattle, which are typically less likely to exhibit clinical disease than sheep. Another possible explanation is that the affected areas do not have high densities of livestock, with most sheep farming occurring in the north and west of England and in Wales, and lower cattle density in the east. Finally, as BTV entered the United Kingdom relatively late in the season, it may be that the infection prevalence in local *Culicoides* populations was low, resulting in a low force of infection.

There is no evidence to suggest that BTV-3 successfully overwintered in the United Kingdom from 2023 to 2024. Vector monitoring determined that the seasonal vector low period (SVLP, defined as fewer than five parous females in a single night’s trap catch across all monitored sites, as per EU legislation) ended on 18 March 2024 (APHA 2024b), earlier than that detected in previous years. However, BTV-3 was detected again in coastal areas of Norfolk and Suffolk in August 2024. With a lack of recent animal movements onto affected holdings, this was once again indicative of wind-borne infected midges coming from the continent (APHA 2024a). The first case was that of a sheep in Norfolk with clinical signs of bluetongue. During the 2024 season, 265 holdings reported animals infected with BTV-3. An “East of England” restricted zone was in place, with active monitoring along the zone border, resulting in regular expansion of the border westwards. Throughout 2024, the clinical picture remained unremarkable, yet reproductive complications were reported early in 2025 during calving and lambing, including abortions and calves born with neurological signs (Swinson et al. 2025).

On 7 February 2025, a single case of BTV-12 was detected on a cattle farm in Kent (where animals infected with BTV-3 were also detected), but no further infections with BTV-12 have been found since. On 1 July 2025, the whole of England became a restricted zone, and on 11 July 2025, the first case of BTV-3 for the new vector season (which began on 23 March 2025) was detected. The year 2025 saw a high proportion of cases from the West Country, particularly Cornwall, where BTV-8 has also been detected. At the time of writing, for the 2025 season, 248 holdings with BTV-3 infections have been reported in England, 1 holding with BTV-8 and 7 holdings with both BTV-3 and BTV-8 (not within the same individual animal). Wales has reported 21 holdings with BTV-3 infections, and Scotland remains free from BTV.

In late November 2025, BTV-3 was detected through routine abattoir surveillance for the first time on a dairy farm in Northern Ireland, with 44 infected animals (RHWG 2025). Subsequently, BTV-3 infection has been detected in four additional herds in the east of Northern Ireland with contiguous 20 km Temporary Control Zones in place in January 2026 (DAERA 2026). Recent reports in January 2026 confirm the detection of BTV-3 in County Wexford, Republic of Ireland (IFA 2026), which, along with the presence of BTV antibodies in cattle in closed herds in the Isle of Man (geographically intermediate between Great Britain and Ireland) (IOM, 2026), suggest that the BTV-3 incursion into Ireland was via infectious wind-borne *Culicoides*.

### Belgium

Belgium remained free of BTV for several years, despite the re-emergence and circulation of BTV-8 in France from 2015 onwards (Sailleau et al. 2017). In February 2019, BTV-8 was identified in Belgium during active winter surveillance. Only a small number of neighboring holdings were affected (AFSCA 2019). Sporadic detections persisted until February 2021, after which no further cases were identified; Belgium was again considered BTV-free in June 2023, according to official veterinary authority communications (Van Leeuw et al. 2025).

In October 2023, BTV-3 was detected in Belgium. The initial outbreaks were located in the northern part of the country, close to the border with the Netherlands. Circulation during the 2023 vector season remained limited, but these detections nevertheless resulted in the loss of Belgium’s BTV-free status and the implementation of control measures (Van Leeuw et al. 2025). In 2024, BTV-3 caused a nationwide epidemic in Belgium. The first confirmed case was detected in early July 2024. Following this initial detection, virus circulation increased rapidly. Within a few weeks, all Belgian provinces were affected, indicating widespread transmission (Van Leeuw et al. 2025). By mid-September 2024, when the Belgian authorities discontinued systematic confirmatory testing of clinically suspected cases, 2,903 affected holdings had been confirmed by RT-PCR. These comprised 1,824 cattle herds, 1,021 sheep flocks, 39 goat holdings, 17 alpaca holdings, and 2 captive cervid holdings. As confirmatory testing was no longer mandatory thereafter, these numbers likely represent a substantial underestimation of the true extent of virus circulation during the epidemic peak (Van Leeuw et al. 2025).

The outbreak was associated with significant clinical and socioeconomic impacts, particularly in small ruminants. National analyses estimated an excess mortality of approximately 92% in small ruminants, corresponding to ∼24,500 additional deaths between July and December 2024. In cattle, excess mortality was estimated at approximately 30%, corresponding to ∼21,100 additional deaths over the same period. Reductions in milk production and in the number of newborn calves were observed at the national level, while movement restrictions and trade limitations further contributed to the overall economic impact (Van Leeuw et al. 2025).

In addition to mortality and production losses, reproductive impacts were documented. Using the mandatory nationwide bovine abortion surveillance system in Belgium, bovine abortion cases reported in 2024 were analyzed and aborted foetuses were tested by RT-PCR for BTV. The first BTV-3 positive aborted foetus with congenital central nervous system malformations was detected in mid-August 2024, followed by a marked increase in abortion-associated congenital malformations later in the year. These findings, and those observed in the United Kingdom, provide field evidence for vertical transmission of BTV-3 (Delooz et al. 2025). Reproductive effects were also described in small ruminants. In naturally infected rams, BTV-3 RNA was detected in semen, accompanied by temporary changes in semen quality, highlighting potential implications for sheep breeding (Martinelle et al. 2025).

In 2025, BTV-3 circulation in Belgium declined markedly compared to 2024, with only sporadic cases detected. However, in late August 2025, the first BTV-8 cases were identified in herds located in the province of Namur near the French border. Although additional detections were reported in other parts of Wallonia, the total number of cases remained limited with no evidence of large-scale epidemic activity.

### Switzerland

After 2015, Switzerland did not experience a widespread bluetongue epidemic comparable to the 2007 to 2011 outbreak, but sporadic detections were reported during subsequent years. National surveillance data indicate that BTV-8 persisted at low levels, with 4 BTV-8 positive animals identified in 2020 and the last reported case recorded in autumn 2020.

This situation changed in August 2024, when BTV reappeared in Swiss livestock after more than 4 yr without confirmed cases, with the detection of both BTV-3 and BTV-8. On 29 August 2024, 3 BTV-3 positive animals were reported from 2 sheep farms in the cantons of Jura and Solothurn, denoting the first identification of this serotype in Switzerland. On 31 August 2024, a single bovine infected with BTV-8 was reported from the canton of Vaud, representing the first BTV-8 detection since 2020. Initial surveillance reported 108 BTV-affected holdings by 16 September 2024, increasing to 2,053 by 20 December 2024 and to about 2,100 by early January 2025 (BTV-3 and BTV-8 combined). By early 2025, ongoing BTV-3 circulation resulted in approximately 219 affected holdings in Switzerland reaching a total of 1,153 holdings in the first half of the year and 2,527 in the second half of the year, indicating continued virus activity during that transmission season (FSVO 2026).

The entire country has been designated as a bluetongue restriction zone (FSVO 2024), and protective measures have been enacted regarding the movement and trade of animals with the EU. These precautions involve restrictions on animal movements and adherence to EU harmonized conditions to prevent further spread of the virus. In 2025, given the extensive spread of BTV, the control measures were amended based on 2 scenarios. Scenario A refers to BTV-3 and BTV-8 outbreaks, serotypes considered already endemic and with preventable severe outcomes due to available vaccination; movement restrictions apply only to sick animals. Scenario B refers to the introduction of exotic serotypes and control measures that have the goal of outbreak containment and movement restriction at farm level.

The post-2015 era in Switzerland thus reflects a transition from a period of minimal bluetongue incidence to a more dynamic situation marked by sporadic low-level cases, re-emergence of multiple serotypes, and renewed control efforts, underscoring the importance of continued surveillance and adaptive vaccination strategies.

## Vaccination Control Programmes

During the 2024 BTV-3 epidemic, three inactivated BTV-3 vaccines were used in northern Europe: Syvazul BTV-3 (Syva), Bultavo-3 (Boehringer Ingelheim) and Bluevac-3 (CZ Vaccines). These vaccines were not fully authorized at the EU level but were deployed under temporary emergency authorizations in accordance with EU legislation (Article 110 of Regulation (EU) 2019/6). Vaccines became available for use in the Netherlands and Belgium in spring 2024 (Van Leeuw et al. 2025), in France in August 2024 (DGAL 2024) and in the United Kingdom in September 2024 (DEFRA 2024). In Switzerland, Bultavo-3 was authorized for use in July 2025, and additional unauthorized vaccines imported under exceptional regulatory measures can be distributed and used until stocks are exhausted or the expiration date is reached (FSVO 2025). In addition, BTVPUR, a multivalent vaccine against serotypes 1, 2, 4, and 8 (although only contains a maximum of 2 serotypes, selected according to the epidemiological situation), was also authorized for use in Switzerland and France. Initially, vaccination across all 5 countries was voluntary, with the available vaccines differing in primary vaccination schedules and claimed efficacy. The vaccines did not claim to induce sterile immunity but aimed to reduce clinical signs and viraemia. At the time of deployment, only limited data on safety and efficacy were available, and no comparative field studies between the vaccines had been conducted. As a result, vaccination in 2024 had a limited protective impact against BTV-3 infection at the population level.

In response to these limitations and the severe economic impact of the 2024 epidemic, Belgium implemented a mandatory vaccination campaign in 2025, targeting cattle and small ruminants. Cattle and sheep born before 1 January 2025 were mandatorily vaccinated against BTV-3 and BTV-8. During the 2025 vector season, surveillance and export testing data indicated markedly reduced virus circulation compared with 2024, with only sporadic detections of BTV-3 and BTV-8.

Several studies have shown that BTV-3 had a high impact in terms of morbidity, mortality and economic costs in 2023 and 2024 (Santman-Berends et al. 2024, van den Brink et al. 2025). Studies under field conditions showed that vaccination with inactivated vaccines in 2024 decreased the impact of BTV-3 in both sheep and cattle in the Netherlands (Dijkstra et al. 2025, Everts et al. 2025).

In France, vaccination against BTV-3 and BTV-8 is voluntary and vaccine doses may be paid for by the Ministry of Agriculture. In Switzerland, vaccination is strongly encouraged and is subsidised by the federal government. As of January 2026, between 6% and 10% of the breeding stock of the United Kingdom had been vaccinated.

## Disease Surveillance

### The Netherlands

To regain BTV-free status, several active monitoring programmes based on EU legislation started in 2009. First, a serological surveillance programme was initiated based on a detection prevalence of 2%. In twenty regions in the Netherlands, 3,000 cattle (150 per region) were serologically tested annually. *Culicoides* monitoring was carried out at 21 farms (those where the serological survey was performed) to determine population dynamics, SVLP and possible overwintering of the vectors. Since all serological samples tested negative between 2009 and 2012, the Netherlands became officially BTV-free again in February 2012. Afterwards, the official BTV-free status was maintained by annual surveys to detect a prevalence of 20%, with 300 cattle sampled in 20 regions (15 per region).

From 1 July 2025, the government changed the notification system of BTV to a monitoring system to detect the spread of BTV and to identify new serotypes in the event of an introduction, although suspicions of bluetongue should still be notified to the official authority. Therefore, 21 veterinary practices in 20 regions of the Netherlands were requested to submit blood samples for RT-PCR from sheep, cattle, and goats with clinical signs of bluetongue. Each veterinary practice could submit samples from a maximum of three animals per holding and a maximum of 14 holdings per region could be included. This system was applied in July, September and November of 2025 and did not show any positive cases of BTV in 2025. In 2026, the same surveillance system is being implemented.

### France

In mainland France and Corsica, BTV serotypes 3, 4 and 8 are considered enzootic. Outbreaks must still be reported, but this does not lead to any specific disease management measures. Notably, animals from outbreak areas can move freely within the country. In the event of trade with EU countries, or export to third countries, health conditions for movement must be met, such as vaccination, testing and/or insect elimination from herds (DGAL 2026).

### UK

After the occurrence of BTV-8 in the Seine Maritime department in France in 2017, the United Kingdom was deemed to be at risk of an incursion event (Grace et al. 2020). Following the requirement for EU member states to conduct active surveillance in the event of elevated risk levels, surveillance was implemented along the south coast of England (Grace et al. 2020). This has been conducted annually in winter since 2017, and it is through this system that BTV-3 was first identified in the United Kingdom in 2023 and BTV-12 in 2025. For winter 2025 to 2026, the purpose of this surveillance was to detect serotypes other than BTV-3 in at-risk counties along the south coast of England. Surveillance is conducted through RT-PCR testing of blood samples taken from randomly selected cattle. As well as identifying BTV-8 at 2 sites in Cornwall and 1 on the Isle of Wight, surveillance has detected further cases of BTV-3 in every county along the south coast of England. Wales and Scotland commenced border surveillance in mid-November 2025, and in Scotland, there have been no detections of BTV. Northern Ireland increased surveillance effort in 2024, in line with EU requirements. Surveillance was increased 4-fold across Northern Ireland and 20-fold along the east coast to account for the risk of wind-borne incursions from Great Britain. Cattle were tested at abattoirs and positive serological results were followed up with testing by RT-PCR, resulting in the detection of 2 BTV-3 positive animals in November 2025.

### Belgium

Annual winter screening is conducted during the SVLP to assess virus circulation that may have occurred during the preceding vector season. Winter screening is normally performed in cattle only and aims to support the demonstration of freedom from specific BTV serotypes and the recovery or maintenance of BTV-free status. The scope and objectives of the winter screening programme may vary according to the epidemiological context. For example, during the 2024 to 2025 winter season, the programme was expanded to include both cattle and sheep, with the objective of assessing BTV-3 seroprevalence following the 2024 epidemic and supporting freedom from other serotypes (BTV-8/BTV-12). Winter screening is primarily based on serological testing by ELISA, and ELISA-positive samples are subsequently tested by RT-PCR, including serotype-specific assays where appropriate, to investigate potential virus circulation.

From 2011 until the end of 2024, Belgium operated a national bovine abortion surveillance programme, aimed at identifying infectious causes of abortion. Within this framework, foetuses presenting congenital lesions were tested for BTV using RT-PCR on foetal tissues, allowing detection of BTV-associated transplacental infections.

### Switzerland

Switzerland has implemented a comprehensive strategy to control bluetongue, which includes surveillance, vaccination policies, financial support, vector control, and regulatory measures. The Federal Food Safety and Veterinary Office (FSVO) continuously monitors the occurrence of the disease and vector activity. Clinical suspect cases are reported to the cantonal veterinary office. Confirmed cases (at farm level) and their evolution are updated regularly on the animal disease dashboard of the FSVO. Annual surveillance for BTV has been carried out yearly (with the exception of 2013 and 2024), and, in 2025, the goal was to determine regional prevalence in cantons with few reported cases. Surveillance is not planned for 2026 due to the downregulation of BTV (EU 2026).

## Vector Surveillance

### The Netherlands

The Centre for Monitoring of Vectors (CMV) performs *Culicoides* monitoring to gather information about the spatiotemporal distribution of *Culicoides* species across the country and for specific habitat types. Monitoring can also be set up for pathogen detection or outbreak surveillance such as during the BTV outbreaks. Some of these data have been published (Takken et al. 2008, Meiswinkel et al. 2014, Mignotte et al. 2020, Uiterwijk et al. 2026) and have been used to create *Culicoides* distribution maps, along with contributions from other countries (VectorNet 2025).

Following the introduction of BTV-3, vector surveillance was carried out at 5 locations where BTV-3 had been diagnosed in ruminants. The 6 main ruminant-associated *Culicoides* species were the most abundant at these farms, and BTV was detected in 5 of those species: *Culicoides chiopterus* Meigen, *Culicoides dewulfi* Goetghebuer, *Culicoides obsoletus* Meigen, *Culicoides punctatus* Meigen and *Culicoides scoticus* Downes and Kettle. In total, 40.5% (155/383) of pools tested positive for BTV, with a mean minimum infection rate (MIR) of 1.4% and an infection rate (IR) of 2.2% (95% CI, 1.9% to 2.6%) (Uiterwijk et al. 2026). The high prevalence of BTV in midges could partly explain the rapid spread of the virus throughout the Netherlands. During this outbreak, no entomological surveys were carried out to determine SVLPs. Instead, to facilitate trade, several Vector Protected Establishments (VPEs) were set up that incorporated entomological monitoring, as described in the delegated regulation (EU) 2020/689 (EU 2019).

### France

A regional vector surveillance network was established in Corsica in 2002/2003 to study *Culicoides* populations dynamics, with a focus on *C. imicola*. Surveillance along the Mediterranean border of mainland France was also set up to detect introductions of *C. imicola* (Baldet et al. 2005). The first entomological surveillance network for *Culicoides* populations covering both continental France and Corsica was set up in 2009, following the emergence of BTV-8 and BTV-1 in mainland France. This surveillance ended in 2012 following the resumption of BTV-free status in mainland France and most of its European neighbors (Hammami et al. 2025). Between 2009 and 2012, surveillance was conducted using OVI UV light suction traps (160 in total, 1-2 per department), monitored weekly from February to April and from October to December to capture the start and end of the SVLP.

With the re-emergence of BTV-8 in 2015, the network was redeployed. During the winter of 2015 to 2016, 49 sites were monitored, and from 2016 to 2018, 24 sites were monitored weekly from October to May. Between 2017 and 2019, various surveillance systems were in place: (i) continental surveillance to determine areas that are seasonally free of all species of *Culicoides*; (ii) island surveillance in Corsica to monitor the long-term dynamics and abundance of populations and to describe the circulation of *Orbivirus* serotypes on a Mediterranean island; (iii) ad hoc surveillance in the Pyrénées-Orientales, and the Var and border area of the Alpes-Maritimes, to monitor the northern distribution limit of *C. imicola*; and (iv) at the request of the ministry, surveillance of the port area of Sète, the point of departure for cattle exports.

Current *Culicoides* surveillance operates across 23 sites with bimonthly collections. This is anticipated to continue until 2027.

### UK

Surveillance for *Culicoides* in the United Kingdom has been running since 2006, following the incursion of BTV-8 into northern Europe (Searle et al. 2014). OVI UV suction traps are run for 1 night per week, with a single trap placed outside at each site. The number of sites has fluctuated over the years, from 7 up to the current network size of 20 sites. The current sites are a mixture of dairy, beef, and sheep farms, and zoos. Trapping takes place from the start of October until SVLP is determined and resumes at the end of February to capture the end of the SVLP. Trapping is paused over the summer. Following the detection of BTV-3 in Great Britain, Northern Ireland ran 7 traps, with each trap located within a geographically spaced 45 × 45 km tetrad.

Following the detection of BTV-3 in Kent in 2023, additional vector surveillance was set up at the IP and at a nearby zoo. One outdoor and 2 indoor *Culicoides* traps were run twice weekly at the IP to determine the start of the SVLP, alongside national vector surveillance. Vector surveillance indicated that the SVLP started on 8 January 2024 (APHA 2024c), the latest start date since vector surveillance began in 2006. In Northern Ireland, increased vector surveillance at the IP was implemented in December 2025, with 1 indoor and 1 outdoor *Culicoides* trap operated 3 times a week until January 2026.

### Belgium

Routine entomological surveillance of *Culicoides* spp. is not conducted as part of the national bluetongue surveillance programme in Belgium. Intensive *Culicoides* monitoring programs assessing the abundance and species diversity in Belgium were undertaken following the BTV-8 outbreak of 2006 to 2008, but systematic monitoring has not been performed since 2012 (Sohier et al. 2018). This monitoring established that multiple *Culicoides* species are present in Belgium, and that members of the *Obsoletus* complex are highly abundant.

### Switzerland

From October 2007 to 2009, the Swiss Federal Veterinary Office initiated a monitoring programme to study *Culicoides* seasonal activity. A total of 19 traps were deployed in 16 designated “bluetongue zones” within Switzerland, including the Principality of Liechtenstein. Specimens were collected on a weekly basis, counted, and taxonomically identified (Kaufmann et al. 2009). Investigations on the occurrence of *Culicoides* at elevated altitudes (Tschuor et al. 2009; Paslaru et al. 2021), as well as on their vector competence (Paslaru et al. 2018), have been carried out.

A study to evaluate the SVLP in the warmest area of the country (Ticino) was conducted from February to April 2025 (Veronesi, E., in preparation), where already on 10 February, 16 parous *Culicoides* were collected, confirming the absence of a sustained SVLP in this part of the country. The Institute of Microbiology of the University of Applied Sciences and Arts of Southern Switzerland (SUPSI), in collaboration with the Institute of Parasitology of the University of Zürich, is running a 3-yr study (2025 to 2028) on the SVLP (funded by the Federal Food Safety and Veterinary Office). Ten farms across three cantons (Ticino, Vaud, and Zürich) are monitored indoors and outdoors from October to April to investigate the impact of indoor and outdoor temperature on *Culicoides* abundance during the coldest months of the year.

## BTV Preparedness

In the United Kingdom, throughout the spring, summer and autumn, risk assessments are conducted to determine the risk of BTV incursion into southern and eastern coastal counties of England through wind-borne transport of infected midges from the continent (Burgin et al. 2013, Grace et al. 2020). These typically take place on a fortnightly basis but increase to weekly during periods of higher risk (most recently the summer months of 2024 and 2025). Incursion risk is assessed for the previous fortnight using disease reports from the near-continent, vector surveillance data, regional temperature data and modelling of *Culicoides* wind-borne dispersal from continental Europe (Burgin et al. 2013). Risk assessment reports are made available online (APHA 2025) and are used to inform preparedness for disease events. Since the spread northwards of EHDV in France in 2023, these risk assessments also include a separate assessment of wind-borne introduction of EHDV.

In the Netherlands, every 4 wk specialists at Wageningen Bioveterinary Research, Royal GD, NVWA and Ministry of LVVN discuss the current situation, lab results and questions from farmers and veterinarians regarding BTV. In case of unexpected clustering in time or region, additional measures may be applicable.

## Advances in Research of BTV Transmission and Control Since 2015

This section provides a synthesis of research studies that have been published since 2015 on vector competence, *in vivo* transmission studies, transmission via germinal products, vaccination, and vector control that are relevant to BTV transmission in northern Europe. For relevant research prior to 2015, Carpenter et al. (2015) critically examined research into vector competence, highlighting the challenges of working with *Culicoides*, as well as the complex factors that determine both vector competence and vectorial capacity. A synopsis of previous research into BTV-host–vector interactions has been provided by Coetzee and Venter (2015), with vector control techniques summarized and critically discussed by Harrup et al. (2016).

### Vector Competence

Vector competence studies in northern Europe are hindered by the current inability to colonize the relevant known and putative vector species (*C. obsoletus*, *C. scoticus*, *C. chiopterus*, *C. dewulfi*, *Culicoides pulicaris* Linnaeus and *C. punctatus*). This results in reliance on field-caught *Culicoides* for infection studies, which do not readily feed in the laboratory. To investigate the potential mechanisms of vector competence, it is often necessary to use two species that have been successfully colonized. These are the model vector species, *Culicoides sonorensis* (Wirth and Jones), which is the primary vector for BTV in North America, and *Culicoides nubeculosus* (Meigen), a northern European species which is not considered to have a role in BTV transmission. The full genome of *C. sonorensis* has been sequenced, and gene expression analysis identified 165 genes that were differentially expressed between BTV competent and refractory individuals (Morales-Hojas et al. 2018). This has provided an indication of the genetic determinants of vector competence for BTV, but full genome sequencing and transcriptomics for northern European vectors of BTV are required to determine if the findings are unique to *C. sonorensis*.

A recent study that used visualization of BTV infection within the salivary apparatus of *Culicoides* using three-dimensional immunofluorescence confocal microscopy revealed the accessory glands as a primary site for BTV replication (Guimerà Busquets et al. 2023). In orally infected *C. sonorensis*, high quantities of the BTV genome within the body were correlated with the presence of BTV in the salivary apparatus. BTV was detected throughout the cell layer of infected accessory glands, compared to localized foci, if detected at all, within the main salivary glands (Guimerà Busquets et al. 2023). The salivary accessory glands of female *Culicoides* are linked via the salivary duct to the main gland and appear to be unique within haematophagous insects (Megahed 1956). Using the same methodology, visualization of the infection dynamics of BTV within the midgut of *C. sonorensis* illustrated infection occurred in foci of midgut cells and could be detected from as early as 2 d post infection (Brown 2024). The impact of subsequent bloodmeals on virus replication, following an infectious bloodmeal was investigated in laboratory colonies of both *C. sonorensis* and *C. nubeculosus* (Brown 2024). This study demonstrated that virus titers increased with additional non-infectious bloodmeals, although the extent of this was dependent on virus serotype and *Culicoides* species. Experimental oral infection studies in *C. sonorensis* found greater infection rates in midges that had fed on BTV-3-infected bloodmeals compared to BTV-8 (Zeiske *et al.* 2025). Infection rates in *C. sonorensis*, however, may not be reflective of those in endemic northern European *Culicoides* within which these serotypes currently circulate.

Field studies have also provided insights into vector competence of different species of *Culicoides* for BTV in northern Europe. *Culicoides* collected from pre-alpine regions in Switzerland showed variable evidence of vector competence to BTV, with *C. scoticus* showing a high virus dissemination rate of 22.5% for BTV-1, comparable to that observed in *Culicoides bolitinos* Meiswinkel and *C. sonorensis* (Paslaru et al. 2018). During the BTV-3 outbreak in 2023 and 2024, *Culicoides* were collected from the field in the Netherlands. Of these, 40.5% of pools tested positive for BTV by RT-PCR. BTV was identified from *C. obsoletus*, *C. scoticus*, *C. chiopterus*, *C. dewulfi*, and *C. punctatus* (Uiterwijk et al. 2026). The high rates of BTV detection in these samples are suggestive of relatively high vector competence and may explain the rapid spread of BTV-3 across the Netherlands and northern Europe.

### *In vivo* Transmission Studies in Animals

Veronesi et al. (2020) demonstrated that simultaneous co-infection of sheep with BTV-1 and BTV-8 results in highly variable transmission and disease outcomes. Four sheep were simultaneously infected with BTV-8 and BTV-1 by *C. sonorensis*, which had been inoculated intrathoracically. Co-infections with both serotypes were established in all sheep subsequent to challenge by *Culicoides* feeding. Unexposed *Culicoides* were then fed directly on the sheep to assess infection rates. Both serotypes persisted in the host, and infection of *Culicoides* was dependent on feeding when the viruses were present in the sheep at sufficient levels, with a dose-dependent relationship for the specific serotype. Clinical severity varied markedly among sheep and was not directly correlated with the number of infected vectors feeding. Occasional mixed infections in vectors highlight the potential for reassortment. Overall, the results emphasize the complexity of BTV transmission dynamics under natural infection routes and underline the importance of serotype-specific surveillance and full genome characterization when multiple strains co-circulate.

Following the re-emergence of BTV-8 in France in 2015, an *in vivo* study was conducted to compare the clinical impact of the new 2015 strain in British sheep with that of the BTV-8 strain previously circulating in 2006 to 2010 (Flannery et al. 2019). Sheep inoculated with a BTV-8 strain isolated from France in 2017 exhibited reduced viraemia (2.9 to 7.9 log_10_ genome copies/ml) compared to those inoculated with the 2007 BTV-8 strain (6.0 to 8.8 log_10_ genome copies/ml). BTV transmission from sheep infected with the re-emergent strain to a model vector species, *C. sonorensis*, fed directly on the animals at peak viraemia, was significantly lower than for the 2007 strain. Clinical impact was also reduced in sheep infected with the 2017 BTV-8 strain, with a greater proportion of sheep exhibiting only mild or no clinical disease compared to those infected with the 2007 BTV-8 strain (Flannery et al. 2019).

Sanders et al. (2022) demonstrated that following genome reassortment, BTV strains may exhibit vector transmission phenotypes of ancestral strains, while the pathogenicity in sheep of reassortant strains was unpredictable. No specific pattern of gene segment inheritance explained the severity of clinical disease within sheep infected with different BTV strains, consistent with previous studies attempting to identify viral genetic determinants of bluetongue severity. Neither of the 2 reassortant BTV-4 strains inherited the full virulence of their ancestral BTV-1 strain, despite sharing 6 or 4 genome segments derived from BTV-1 (Sanders et al. 2022). Acute presentation of sheep infected with an ancestral BTV-4 strain was milder than that observed in sheep infected with the 2 reassortant strains. However, the chronic presentation of disease led to a similar overall clinical score between the strains. In contrast, differences in the infection rate of *C. sonorensis* fed on sheep infected with each BTV strain were consistent. *Culicoides sonorensis* was close to refractory for infection with the ancestral BTV-1 and BTV-8 strains but exhibited significantly higher infection rates for the ancestral BTV-4 strain and reassortant BTV-4 strains. The study supports the hypothesis that rapid changes in severity of clinical outcome in ruminants and transmission in *Culicoides* may result from reassortment of co-circulating BTV strains.

An investigation of the clinical impact and infection kinetics associated with the infection of UK sheep with the emergent BTV-3 strain was conducted in 2024 (Newbrook et al. 2025). North County mule sheep were infected with a UK BTV-3 strain isolated from a cow infected in 2023. To initiate infection, sheep were exposed to the bites of infected *C. sonorensis*. All exposed sheep developed a viraemia, with the BTV genome detected from 2 dpi. All BTV-3-infected sheep exhibited mild to moderate signs of bluetongue, characterized by fever, lameness, haemorrhagic diarrhoea, and depression/reluctance to move. Clinical signs and severity were highly variable between individual sheep. Three sheep were euthanized on reaching humane endpoints, one recovered from extended fever following treatment, and the fifth animal exhibited a mild, short fever and no further signs of disease. BTV was isolated up to 28 dpi from one of the surviving sheep. The study demonstrated that although clinical disease in sheep had not been seen in the United Kingdom prior to the study, sheep infected with BTV-3 in the United Kingdom were likely to show variable clinical signs and severity of disease (Newbrook et al. 2025).

### Transmission via Germinal Products (BTV-8)

Belgian experimental studies have shown that BTV-8 can be transmitted via germinal products, extending the current understanding of BTV transmission. In a controlled experimental setting, artificial insemination with frozen semen collected from naturally infected bulls resulted in viraemia and seroconversion in susceptible heifers despite absent or only mild clinical signs and was associated with early pregnancy loss in a subset of animals. Differences in viral load between semen batches had a significant impact on infection outcome and the timing of viraemia, indicating a dose-dependent transmission process (De Clercq et al. 2021).

Complementary studies investigated the potential for BTV-8 transmission via embryo transfer. In an initial *in vitro* approach, both *in vitro*—produced and *in vivo*—derived bovine blastocysts were experimentally exposed to BTV-8 and subjected to standard and intensified washing and trypsin treatment protocols, following International Embryo Transfer Society (IETS) recommendations. Viral RNA remained detectable in both embryo types after these procedures. Transmission to recipient cows was subsequently demonstrated by the development of viraemia and seroconversion following embryo transfer of *in vivo*—derived BTV-8—exposed embryos processed according to routine IETS recommendations (Haegeman et al. 2019). These findings indicate that standard embryo processing may not fully prevent BTV-8 transmission and that transmission risk may depend on both viral strain and embryo type.

Together, these studies highlight that BTV-8 exhibits an atypical transmission phenotype compared to most other BTV serotypes, providing a plausible mechanism for virus persistence and re-introduction independent of vector activity. This has direct implications for the regulation of germinal product movements, surveillance strategies and risk assessment during and after bluetongue outbreaks.

### Vaccination

Only inactivated vaccines are allowed within the European Union due to their safety. While these vaccines are available for several serotypes and have proven crucial for controlling recent outbreaks, they are generally serotype-specific, and cross-protection between serotypes remains limited and often incomplete (Bréard et al. 2015, Martinelle et al. 2018, Fay et al. 2021). Live-attenuated vaccines present the risk of “reversion-to-virulence” (Veronesi et al. 2010), transmission by *Culicoides* (Veronesi et al. 2005) and reassortment with circulating viruses in the field (van Rijn 2019). The disabled infection single animal (DISA) vaccine is a potential novel platform, based on a deletion in BTV segment 10 introduced via reverse genetics (van Rijn et al. 2017, van Rijn et al. 2021a, van Rijn et al. 2021b). This platform has been used in both sheep and cattle and provides sterile immunity upon challenge with the virus. Also, multiple serotypes can be combined into a single vaccine dose by exchanging viral proteins 2 and 5 from different serotypes on a different backbone. Additionally, the DISA-virus does not disseminate back in midges from the midgut to the salivary gland and is compliant with the differentiating infected from vaccinated animals (DIVA) principle (Feenstra et al. 2015, Tacken et al. 2015).

### Vector Control

Vector control strategies for *Culicoides* are currently limited, partly due to incomplete knowledge about the ecology of *Culicoides* populations and partly due to the presence of a wide variety of different breeding habitats on and around farms. Indeed, it is not known to what extent the *Culicoides* population must be controlled in order to stop outbreaks. The 4 recognized Palaearctic vector species in the *Avaritia* subgenus, particularly *C. dewulfi* and *C. chiopterus*, are known to use cattle dung as a larval development substrate, but a study that restricted *Culicoides* access to dung heaps on farms showed no significant impact on the local population of *Avaritia* (Harrup et al. 2014). Harrup et al. (2016) noted the importance of *Culicoides* control techniques in the absence of safe, efficacious vaccines or when it is not economically viable to deploy such vaccines.

Recent studies have assessed the efficacy of vector-proofing animal housing to reduce contact between *Culicoides* and susceptible hosts. Insecticide-treated screening on horse stable doors was shown to reduce the entry of *Culicoides* (Baker et al. 2015). In response to the BTV-3 outbreak in the United Kingdom, King et al. (2025) found that animal housing can be easily fitted with readily available vector-proof materials to significantly reduce vector-host contact. The VPEs set up to facilitate trade in the Netherlands have been shown to be highly effective at excluding *Culicoides* and protecting against BTV. During the BTV-3 outbreak in the Netherlands, there was evidence to suggest that the use of fans and good ventilation in animal housing reduced the transmission of BTV. Indeed, horizontal ventilation in stabling was previously shown to reduce BTV-8 transmission in dairy cattle (Santman-Berends 2011).

In general, repellents and insecticides are not advised for the protection of livestock against BTV due to their ecotoxicity, short-lived efficacy, limited coverage and, for insecticides, their delayed action (EFSA 2017). There is no evidence to suggest that they are able to reduce BTV transmission in the field.

## Climate Change

Circumstantial evidence suggests that the emergence of BTV in Europe was linked to regional warming over the corresponding time period because: (i) there was an increase in temperatures in outbreak areas; (ii) multiple strains spread from multiple sources at the same time; and (iii) there were few, if any, changes to livestock management and land-cover (Purse et al. 2005, Purse et al. 2015).

Genetic evidence suggests that *C. imicola* was already well established prior to the first reported BTV outbreaks in Cyprus in 1924 (Jacquet et al. 2015). Accordingly, recent climate change is unlikely to be responsible for the introduction of this species to the region. Monitoring of *C. imicola* populations in the Var and Alpes Maritimes departments in mainland France (Mediterranean region) was carried out every year between 2003 and 2019, with the exception of 2016 and 2018, to determine the abundance and geographic distribution of the northernmost population of *C. imicola* in Europe. More than 90 sites were sampled over the period and *C. imicola* was captured at least once in 68 of them. The populations are concentrated on the plains and on the coast, at altitudes not exceeding 250 m above sea level. These data, representing 15 yr of monitoring, show that the northern distribution front in the Mediterranean basin does not appear to be changing (Venail et al. 2012, Jacquet et al. 2016). These populations remain the northernmost of the known distribution of *C. imicola* in the Western Palearctic region.

In 2021, a more detailed study of the spread of *C. imicola* on the continent was carried out to determine whether this species had been able to spread eastwards towards the Italian border and westwards toward the Bouches-du-Rhône departments in low-altitude areas. This found that the distribution of *C. imicola* had not expanded since the first captures made in 2003 and remained confined to the coastal area bordering the eastern Var and western Alpes-Maritimes departments. The Argens valley remains the most favorable area for this species. However, the abundance of *C. imicola* was higher in 2021 than during the two previous collection years of 2015 and 2017 and was associated with more favorable weather conditions.

Changes in populations of Palearctic species over a 40-yr period have been assessed at 2 sites in England (Sanders et al. 2019). This identified an increase in abundance and an increase in the length of the seasonal activity period at 1 site, but not at the other, driven by changes in temperature, precipitation, and soil moisture. In general, higher temperatures result in greater *Culicoides* abundance (up to a point), if all other factors are also suitable (eg humidity, host availability, breeding site availability). BTV outbreaks require both the presence of the virus and suitably abundant vectors, but it is not known what this combined tipping point is and to what extent it is temperature driven.

Phylogeographic analyses of 113 BTV outbreaks between 1998 and 2012 did not identify a significant role of temperature in the expansion of BTV (Jacquot et al. 2017). However, the authors suggest this could be a consequence of either methodological (eg they assumed a linear response to temperature and used monthly averages) or biological (eg strain variation in the response to temperature, which was not accounted for) reasons. By contrast, several analyses have shown that the basic reproduction ratio (ie the number of secondary cases per primary case in a wholly susceptible population) and, hence, the potential for BTV transmission, increased substantially in the decades after 1990 as a result of changes in temperature (Guis et al. 2012, Baylis et al. 2017, Brand and Keeling 2017). In southern Europe, this was because of a predicted increase in the abundance of *C. imicola*, while in northern Europe it was because of a predicted increase in the ability of Palearctic vector species to transmit BTV, especially through a shortening of the extrinsic incubation period (Guis et al. 2012, Baylis et al. 2017).

Based on predicted changes in temperatures, 2 studies have predicted that the risk of BTV transmission in Europe will increase, in terms of both duration of the transmission season and areas at risk (Brand and Keeling 2017, Jones et al. 2019). However, both studies ignore the potential impacts of climate change on vector abundance and seasonality, as well as land use change on vector and host distributions, which limits the value of the predictions. Moreover, a lack of life history parameters for most European *Culicoides* vector species makes it challenging to predict changes in vector populations (Purse et al. 2015). Advances in machine learning may enable greater insight into environmental drivers of disease, including serotype-specific factors (Alkhamis et al. 2021). However, robust long-term trends in *Culicoides* abundance or distribution remain difficult to assess because entomological surveillance in Europe varies considerably between countries, is limited in several areas, and has changed substantially over time in response to bluetongue outbreaks (EFSA 2017, ECDC 2021). Consequently, while changes in seasonal activity are increasingly evident, their broader epidemiological consequences are more challenging to evaluate.

## Globalization

Wind-borne dispersal of *Culicoides*, either as infected vector incursions or as population range expansion, has been demonstrated, although it remains difficult to quantify (Burgin et al. 2013, Jacquet et al. 2015, Bibard et al. 2024, Bibard et al. 2025). There is evidence that *Culicoides* dispersal across water can be over distances of up to 700 km (Eagles et al. 2014). This and wind-borne dispersal across land over shorter distances both present high-risk pathways for BTV incursions. Genetic population models show high connectivity of *C. obsoletus* across Europe, which allow for a high level of gene flow between populations (Mignotte et al. 2021).

Live animal movements are a known risk pathway for BTV introduction and post-import testing has identified multiple incursions of BTV-infected animals into northern Europe, as discussed above. For those that have not resulted in onward transmission, early detection and tracing, and conditions upon entry (eg location and time of year) have been key. The international trade in live animals has increased significantly over the past 2 decades, which has likely increased the frequency of BTV-infected animals entering northern Europe and moving between countries.

The potential for transportation of immature or adult *Culicoides* via commercial activities (transport of goods, live animals or people) is one that is often raised but rarely studied. The lack of data on the transport of *Culicoides* via human activities can be explained in large part by the difficulty of implementing experimental protocols, which require the collaboration of private partners (transporters) and authorities at border entry points or quarantine areas in border zones. Within Europe, the transport of live animals is often via road, a route that is not monitored for vectors. In the Netherlands, areas near border entry points, such as parking areas along highways, airports, and flower auctions, are monitored for *Aedes albopictus* (Skuse), with traps at several locations suitable for catching *Culicoides*. *Aedes albopictus* monitoring is carried out at some border entry points in the United Kingdom, but the traps used are not suitable for the collection of *Culicoides.*

A recent paper investigated the potential for importing *Culicoides* into Northern Europe with cut flowers produced in East Africa (Stokes et al. 2026). This study showed that the number of *Culicoides* captured on the horticultural farm studied was very low, decreasing along the production chain and reaching zero *Culicoides* in the last production step, which includes cold rooms. There were no *Culicoides* aspirated from flowers, and the authors concluded that there is a very low risk of importation through the cut flower trade. *Culicoides* have been collected from indoor traps located at airports and flower auctions (points of entry), but it was not possible to determine if these individuals were imported. Two studies from eastern Asia have previously assessed the risk of *Culicoides* being transported by sea freight. WeiZhong et al. (2005) inspected 70 ships arriving at Qinhuangdao port in China from neighboring countries (Korea, China, Japan, India) by vacuuming the windows of the cabins inhabited by the crew. A total of 53 insects were collected from 29 (out of 70) ships, with nine *Culicoides* individuals identified. More recently, Kim et al. (2015) conducted entomological monitoring in Korea, on Jeju Island, at locations including an airport cargo warehouse, an animal quarantine station and a port. Very few individuals were collected from these locations, and no exotic species to the expected fauna were found.

The first and only mention of *Culicoides* being transported via commercial air transport dates back to 1964 (Reye 1964) and describes the possible transport of a harmful species of *Culicoides* (aggressive toward humans) between Fiji and the Society Islands by a small tourist plane. Two studies mention an individual *C. imicola* in Switzerland (Ticino) outside the known distribution area of the species (Cagienard et al. 2006, Napp et al. 2013). The authors proposed the hypothesis of introduction via air transport due to the presence of Lugano airport near the collection site, or wind dispersal. However, these hypotheses have now been dismissed in favor of the hypothesis that the trap used was contaminated by material from Corsica.

Two further unpublished studies in Belgium and France provide very little additional data. In Belgium, 7 entry points (air, motorways, and ports) were monitored between April and November 2013, but no invasive species were detected, and abundances were very low (Deblauwe, unpublished). In France, entomological monitoring was carried out at the port of Sète (point of departure for live animals) between 2015 and 2018. Two *Culicoides* were collected in April 2017, and 2 in May 2018, but they likely originated from a nearby farm.

In summary, there is very little evidence to support the frequent and large-scale transport of *Culicoides* individuals via commercial air or sea travel in Europe. There are no studies evaluating the possibility of transporting *Culicoides* with live animals (by road or sea), and, to our knowledge, there are no publications detailing monitoring work in airport areas or directly in aircraft. To date, studies have focussed on adult stages, which represent disease risk, and are easier to capture, with no studies assessing the risk of transport of immature stages.

## Conclusions and Recommendations

Bluetongue virus epidemiology shows clear geographic differences across Europe. In parts of southern Europe, BTV is detected more regularly over time, whereas in north-western Europe, detection has mainly occurred during intermittent outbreak periods, rather than through continuous circulation. The reasons for these differences are not fully understood. Circulation in southern Europe is often discussed in relation to possible repeated virus introductions via wind or animal movements from neighboring regions, including North Africa, where surveillance is more limited. In north-western Europe, the factors leading to virus introduction and establishment remain unclear (Wilson and Mellor 2009). The actual introduction route of BTV strains causing the large-scale BTV-8 outbreak in 2006 to 2008 and the BTV-3 outbreak that began in 2023 remain elusive but are likely the result of a combination of globalization (including animal movements) and climate change. Overall, these patterns highlight important knowledge gaps in the pathways and drivers of BTV emergence across Europe.

Changes in climate, land-use, biodiversity, trade, travel, and related mitigation actions may lead to changes in vector habitat suitability and vector-borne disease distribution. Vector monitoring is an essential component to map spatio-temporal *Culicoides* distribution, and to assess *Culicoides*-borne disease risks. In particular, it will become increasingly important to understand how changes in land use, such as raising ground-water levels (LVVN 2020), rewilding and urbanization will affect both vector populations and vector-host interactions. As well as the potentially direct impacts of climate change on *Culicoides* populations, how humans and livestock adapt to climate change (eg behavior, farm management practices) will have impacts on disease risk.

In January 2026, the EU published its intention to change bluetongue from a category C + D+E disease to a category D + E disease, with effect from July 2026 (EU 2026). The rationale for this decision is cited as an increase in the abundance, persistence, and expansion of vectors over longer periods, a dramatic increase in the distribution of multiple BTV serotypes within the EU and the increased persistence of infection in a wide range of ruminant hosts. It is considered, therefore, that the profile of the disease has changed, and prevention and control measures are limited in terms of feasibility and effectiveness. This change in regulation will likely have consequences in terms of surveillance for both the disease and vectors within the EU.

However, despite the change in disease category for bluetongue within the EU, further research is required. Here, we assert the need for investment in long-term monitoring of *Culicoides* and *Culicoides*-borne viruses across northern Europe due to their potential socio-economic impact and the continued risk of incursion of new serotypes and reassortant viruses. In particular, a greater understanding of species-specific *Culicoides* ecology is required. Further investigations of the overwintering mechanisms that may sustain BTV transmission between years in northern latitudes, such as the winter activity of adult *Culicoides* and duration of infectiousness of ruminants, are needed. Advances in vector control techniques are required, including building on anecdotal evidence, such as the beneficial use of ventilation in animal housing. The inability to colonize northern European *Culicoides* vector species within a laboratory setting presents challenges to both life-history and infection studies. Characterization of the viral, vector, and host determinants of BTV strain emergence, spread and clinical impact within Europe will greatly improve outbreak prediction and response. Finally, there is a need to fund and maintain collaborative platforms for data sharing across Europe to facilitate access for both researchers and policymakers.

## Author Contributions

Marion England (Conceptualization [lead], Data curation [lead], Formal analysis [lead], Investigation [lead], Writing—original draft [lead], Writing—review & editing [lead]), Thomas Balenghien (Data curation [supporting], Formal analysis [supporting], Investigation [supporting], Writing—original draft [supporting], Writing—review & editing [equal]), Carrie Batten (Data curation [supporting], Formal analysis [supporting], Investigation [supporting], Writing—original draft [supporting], Writing—review & editing [equal]), Emmanuel Bréard (Data curation [supporting], Formal analysis [supporting], Investigation [supporting], Writing—original draft [supporting], Writing—review & editing [equal]), Ilse De Leeuw (Data curation [supporting], Formal analysis [supporting], Investigation [supporting], Writing—original draft [supporting], Writing—review & editing [equal]), Nick De Regge (Data curation [supporting], Formal analysis [supporting], Investigation [supporting], Writing—original draft [supporting], Writing—review & editing [equal]), Maxime Duhayon (Data curation [supporting], Formal analysis [supporting], Investigation [supporting], Writing—original draft [supporting], Writing—review & editing [equal]), Claire Garros (Data curation [supporting], Formal analysis [supporting], Investigation [supporting], Writing—original draft [supporting], Writing—review & editing [equal]), Pachka Hammami (Data curation [supporting], Formal analysis [supporting], Investigation [equal], Writing—original draft [supporting], Writing—review & editing [equal]), Melle Holwerda (Data curation [supporting], Formal analysis [supporting], Investigation [supporting], Writing—original draft [supporting], Writing—review & editing [equal]), Archie Murchie (Data curation [supporting], Formal analysis [supporting], Investigation [supporting], Writing—original draft [supporting], Writing—review & editing [equal]), Christopher J. Sanders (Data curation [supporting], Formal analysis [supporting], Investigation [supporting], Writing—original draft [supporting], Writing—review & editing [equal]), Mathilde Uiterwijk (Data curation [supporting], Formal analysis [supporting], Investigation [supporting], Writing—original draft [supporting], Writing—review & editing [equal]), Rene van den Brom(Data curation [supporting], Formal analysis [supporting], Investigation [supporting], Writing—original draft [supporting], Writing—review & editing [equal]), Eva Veronesi (Data curation [supporting], Formal analysis [supporting], Investigation [supporting], Writing—original draft [supporting], Writing—review & editing [equal]), and Simon Gubbins (Data curation [supporting], Formal analysis [equal], Investigation [supporting], Writing—original draft [equal], Writing—review & editing [equal])

## Funding

M.E., S.G., C.B., and C.S. acknowledge funding from UK Research and Innovation Biotechnology and Biological Sciences Research Council (grants BBS/E/PI/23NB0003, BBS/E/PI230002C, BBS/E/PI/23NB0004, BBS/E/PI/230001B, and BBS/E/PI/230002B). M.E., S.G., and C.B. also acknowledge funding from the Department for Environment, Food and Rural Affairs (DEFRA), C17508—Provision of National Reference Laboratory, Disease Surveillance and Outbreak Response Function for Vesicular and Non-Vesicular Disease. E.V. acknowledges funding from CH-3003 Bern contract numbers 714003244 and 714003267. This study was also financially supported by the Dutch Ministry of Agriculture, Fisheries, Food Security and Nature under project WOT-01-002-041 VZVD and the French Ministry of Agriculture, Agri-Food and Food Sovereignty and the General Directorate for Food (DGLA). A.M. is supported by grant-in-aid funding from Department of Agriculture, Environment and Rural Affairs (DAERA), Northern Ireland.

## Conflicts of Interest

None declared.
